# Infant sleep predicts trajectories of social attention and later autism traits

**DOI:** 10.1111/jcpp.13791

**Published:** 2023-03-29

**Authors:** Jannath Begum‐Ali, Louisa K. Gossé, Luke Mason, Greg Pasco, Tony Charman, Mark H. Johnson, Emily J.H. Jones, The STAARS Team, Mary Agyapong, Tessel Bazelmans, Leila Dafner, Mutluhan Ersoy, Teodora Gliga, Amy Goodwin, Rianne Haartsen, Hanna Halkola, Alexandra Hendry, Rebecca Holman, Sarah Kalwarowsky, Anna Kolesnik, Sarah Lloyd‐Fox, Nisha Narvekar, Laura Pirazzoli, Chloë Taylor

**Affiliations:** ^1^ Department of Psychological Sciences, Centre for Brain and Cognitive Development Birkbeck, University of London London UK; ^2^ Psychology Department Institute of Psychiatry, Psychology & Neuroscience, King's College London London UK; ^3^ Department of Psychology University of Cambridge Cambridge UK

**Keywords:** Attention deficit hyperactivity disorder, infancy, autism spectrum disorder, sleep, social attention, eye tracking

## Abstract

**Background:**

Children with neurodevelopmental disorders including autism spectrum disorder (ASD) and attention deficit hyperactivity disorder (ADHD) often experience sleep disturbances, but little is known about when these sleep differences emerge and how they relate to later development.

**Methods:**

We used a prospective longitudinal design in infants with a family history of ASD and/or ADHD to examine infant sleep and its relation to trajectories of attention and later neurodevelopmental disorders. We formed factors of Day and Night Sleep from parent‐reported measures (including day/night sleep duration, number of naps in the day, frequency of night awakenings and sleep onset problems). We examined sleep in 164 infants at 5‐, 10‐ and 14‐months with/without a first‐degree relative with ASD and/or ADHD who underwent a consensus clinical assessment for ASD at age 3.

**Results:**

By 14‐months, infants with a first‐degree relative with ASD (but not ADHD) showed lower Night Sleep scores than infants with no family history of ASD; lower Night Sleep scores in infancy were also associated with a later ASD diagnosis, decreased cognitive ability, increased ASD symptomatology at 3‐years, and developing social attention (e.g., looking to faces). We found no such effects with Day Sleep.

**Conclusions:**

Sleep disturbances may be apparent at night from 14‐months in infants with a family history of ASD and also those with later ASD, but were not associated with a family history of ADHD. Infant sleep disturbances were also linked to later dimensional variation in cognitive and social skills across the cohort. Night Sleep and Social Attention were interrelated over the first 2 years of life, suggesting that this may be one mechanism through which sleep quality influences neurodevelopment. Interventions targeted towards supporting families with their infant's sleep problems may be useful in this population.

## Introduction

Sleep disturbances are one of the most common co‐occurring conditions in children with neurodevelopmental disorders (Cortese, Faraone, Konofal, & Lecendreux, 2009; Cortese, Wang, Angriman, Masi, & Bruni, 2020). Children with attention deficit hyperactivity disorder (ADHD) or autism spectrum disorder (ASD) often experience clinically‐significant sleep problems that include frequent night wakening (Robinson‐Shelton & Malow, 2015), sleep onset problems (Levin & Scher, 2016) or sleep apnoea (Horne, Wijayaratne, Nixon, & Walter, 2019). Sleep problems may exacerbate daytime neurobehavioural symptoms in ASD, appear to be stable in the absence of intervention, may associate with concurrent sensory over‐reactivity and predict the development of later ADHD symptoms (Mazurek, Dovgan, Neumeyer, & Malow, 2019).

Sleep disturbances in children with neurodevelopmental disorders may begin in infancy. For example, in a large birth cohort study the number of parent‐reported night awakenings at 12‐months predicted ASD scores at 2‐years (Nguyen, Murphy, Kocak, Tylavsky, & Pagani, 2018). Further, Huhdanpää et al. (2019) found that severe sleep problems between 6‐ and 12‐months were associated with an increased likelihood of later ADHD. Early sleep differences may also relate to trajectories of brain development: sleep onset problems in the first year were more common in infants with later ASD than TD infants, and related to the development of the hippocampus between 6‐ to 24‐months (MacDuffie et al., 2020). Thus, disruptions to sleep may appear early in the development of children later diagnosed with ASD and ADHD. Sleep disruptions may persist into childhood and adolescence in the form of shorter sleep durations (Humphreys et al., 2014) or parent‐reported presence of sleep problems (Hvolby, 2015; Verhoeff et al., 2018).

Early disrupted sleep patterns have also been linked to social competence (Tomisaki et al., 2018) and poorer overall cognitive development in childhood; though the precise sleep variables that are most informative vary between studies, important measures to capture include napping, night awakenings, night sleep duration and settling parameters (e.g. Astill, Van der Heijden, Van IJzendoorn, & Van Someren, 2012; Field, 2017; Tham, Schneider, & Broekman, 2017 for reviews). In early development, associations between poor sleep (including quality, proportion of night sleep, sleep duration and sleep problems) and disruptions in attention have been commonly observed (Bernier, Beauchamp, Bouvette‐Turcot, Carlson, & Carrier, 2013; Huhdanpää et al., 2019; O'Callaghan et al., 2010; Sadeh et al., 2015). Geva, Yaron, and Kuint (2016) suggest that sleep and attention may be closely related due to their shared underlying brain anatomy, including that the brainstem‐based Ascending Reticular Arousal System shows widespread connections to areas governing sleep (e.g., hypothalamus) and attention (e.g., thalamus). Thus, early differences in sleep in infants with neurodevelopmental disorders could be associated with early disruptions to attention and later effects on cognition and social functioning.

Differences in visual attention have been identified in prospective studies of infants with a family history of ASD and ADHD. Infants with later ASD show declines in attention towards faces within complex social real‐world or screen‐based stimuli with age (Gangi et al., 2021) and slowed non‐social attention disengagement at 14‐months (Elsabbagh et al., 2013). Within static stimuli, longer unbroken looks at 14‐months (a pattern usually seen in younger infants) are associated with lower effortful control in toddlerhood (Hendry et al., 2018), higher polygenic scores for ADHD and more ADHD symptoms in later childhood (Gui et al., 2020). Further, infants with a family history of ADHD show decreased general attentiveness to a screen (Miller, Iosif, Young, Hill, & Ozonoff, 2018). Thus, there is emerging evidence that disruptions in attention are apparent in the early development of children with later ASD and ADHD.

Given that disturbances in sleep and attention are both seen prior to diagnoses of ASD and ADHD, and due to the close link between brain systems underpinning sleep and attention, we reasoned that there may be developmental links between atypicalities in these domains. Thus, we investigated the association between early visual attention, early sleep patterns and later emerging ASD/ADHD symptomatology and cognitive and social adaptive skills in a longitudinal sample of infants with and without a family history of ADHD and/or ASD. Given that ASD has been previously associated with infant night sleep difficulties (e.g., MacDuffie et al., 2020; Nguyen et al., 2018), we predicted that those with an elevated likelihood of ASD (ASD‐L), and those who go on to later develop ASD (ASD+), would demonstrate lower levels of night time sleep, and that lower levels of night sleep would be associated with lower levels of cognitive ability and decreased social functioning (Bernier et al., 2013; Sadeh et al., 2015; Sorondo & Reeb‐Sutherland, 2015; Tomisaki et al., 2018). Within longitudinal models, we examined the relationship between repeated measurements of eye‐tracking measures of attention to social (Face Popout) and non‐social stimuli (Gap‐Overlap), parent‐report measures of day and night sleep and later developmental and clinical outcomes. Given previous work on differences in visual attention in infants with a family history of ASD, we predicted that poorer sleep would associate with less efficient social attention (Gui et al., 2020) and slower disengagement (Elsabbagh et al., 2013).

## Method

### Participants, design and procedure

Participants were recruited for a longitudinal study running from 2013 to 2019. Infants could enrol if they either had a first‐degree relative with a clinical diagnosis of ASD from a licenced clinician, a first‐degree relative with community clinical or probable research diagnosis (using a screening tool from the Conner's family; Conners, 2008; Conners & Goldstein, 2009) of ADHD, or no first‐degree relatives with either diagnosis and a typically developing older sibling (see SM 1.1 and Appendix: SM 5 for full details). Inclusion criteria included full‐term birth (gestational age > 36 weeks), and no known medical or developmental condition. Informed written consent was provided by the parent(s). Ethical approval was granted by the National Research Ethics Service and the Departmental Research Ethics Committee.

For analysis, each infant in the study was assigned a binary rating for the confirmed presence or absence of a first‐degree relative with ASD and ADHD. This approach allowed us to test the effect of elevated/familial likelihood of ASD (ASD‐L), elevated/familial likelihood of ADHD (ADHD‐L), and their interaction, recognising that ASD and ADHD diagnoses often co‐occur (Stevens, Peng, & Barnard‐Brak, 2016). The final sample of 164 participants included 80 infants with a first‐degree relative with ASD only (ASD‐L), 31 infants with a first‐degree relative with ADHD only (+3 half‐siblings with ADHD only; ADHD‐L), 21 infants with first‐degree relatives with both ASD and ADHD (ASD + ADHD‐L) and 29 infants with no family history of either condition (TL; see Table 1 and Table S1 for final demographics).

**Table 1:** Sample demographics table by Elevated Likelihood (EL) and ASD Outcome

	TL	EL	EL‐ASD−	EL‐ASD+
5‐months				
*n*	27	82	54	10
Gender	18m, 9f	44m, 38f	27m, 27f	5m, 5f
Age in days (SD)	180.11 (19.37)	174.26 (18.42)	171.85 (18.42)	173.7 (18.87)
MSEL ELC	85 (9.32)	83.99 (10.51)	85.15 (9.66)	82.6 (15.01)
10‐months				
*n*	27	123	87	12
Gender	16m,11f	66m, 57f	44m, 43f	7m, 5f
Age in days (SD)	321.93 (16.7)	321.09 (17.86)	321.39 (19.05)	322.25 (11.76)
MSEL ELC	88.89 (12.19)	86.88 (15.38)	88.74 (15.69)	85.67 (12.45)
14‐months				
*n*	23	120	88	12
Gender	13m, 10f	68m, 52f	47m, 41f	7m, 5f
Age in days (SD)	447.61 (18.25)	450.73 (22.25)	452.9 (22.38)	441.67 (16.74)
MSEL ELC	78.78 (11.99)	77.48 (12.31)	78.66 (13.13)	69.92 (7.29)
3‐years				
*n*	19	100	86	11
Gender	12m, 7f	52m, 48f	44m, 42f	7m, 4f
Age in days (SD)	1136.79 (12.96)	1146.13 (54.02)	1145.79 (56.47)	1145.64 (41)
MSEL ELC	129.05 (11.75)	110.18 (19.18)	112.45 (18.53)	90.2 (14.55)
SRS total	23.37 (9.46)	43.64 (33.89)	32.82 (20.61)	109.36 (27.32)
CBCL ADHD	3.05 (2.16)	4.59 (3.44)	3.9 (3)	9.91 (1.38)

Three participants with half siblings diagnosed with ADHD were only in analyses examining ASD Outcome, but are reported in the demographics table above. Total participant numbers differ between Elevated Likelihood and Outcome as not all participants attended the 3‐year diagnostic visit. Sample demographics with ASD Outcome are only reported for the elevated likelihood (EL) groups. Elevated Likelihood includes all infants with a family history of ASD and/or ADHD (ASD‐L, ADHD‐L and ASD + ADHD‐L cohorts). EL‐ASD+ indicates participants who were ASD positive at the 3‐year diagnostic visit (ASD+ in the main text), whereas EL‐ASD‐ indicates participants who were ASD negative at this visit (ASD− in the main text). Please note ASD Outcome analyses in the main text also included TL infants.

We examined infant sleep and its associations with later visual attention and developmental outcome phenotypes. We collected measures of infant sleep (via parent‐report questionnaire measures) at 5, 10 and 14 months of age. At these timepoints, participants came in for a day long visit and took part in a battery of tasks, including measures of eye tracking. Following the eyetracking tasks, all other behavioural measures (e.g., the MSEL; Mullen, 1995) were completed. At the 3‐year visit, measures of developmental outcome were also conducted. Parents completed any sleep questionnaires prior to their attendance at the lab. See SM 1.2, SM 1.3 and below for full details of our sleep questionnaires, eyetracking and developmental outcome measures.

### Measures

#### Parent report measures of sleep

The Sleep and Settle Questionnaire (Matthey, 2001) is a 34‐item parent‐report questionnaire that assesses infant sleep and settling behaviour. The SSQ has demonstrated good psychometric properties, with researchers arguing that it shows sensitivity to change/treatment effects and is able to differentiate between infant sleep problems as reported by parents (Lewandowski, Toliver‐Sokol, & Palermo, 2011). We extracted a subset of items: the duration of daytime sleep, daytime nap number, duration of night time sleep and night awakening number (all reported as averages over the previous week). Durations were transformed to minutes and if parents reported a range (e.g., 1–2 naps), the mean value was taken (1.5 naps). Participants were excluded from specific Day/Night analyses if they had missing data for that analysis (e.g., participants were excluded from Day Sleep analyses if they had missing values for day sleep duration or number of day naps). We further extracted the ‘Infant sleep onset problems’ variable (ISOP) from the Infant Behaviour Questionnaire‐Revised (Putnam, Helbig, Gartstein, Rothbart, & Leerkes, 2014) collected at 5‐, 10‐ and 14‐months as this has shown differences in sleep in infants with and without later ASD (MacDuffie et al., 2020; SM 1.2).

#### Measures of visual attention

Visual stimuli were presented, and eye tracking data acquired, using a Tobii TX‐300 eye tracker (SM 1.3.1).

##### *Attention shifting: Disengagement* (Gap‐Overlap task; SM 1.3.2)

Shifts of visual attention were measured using the gap‐overlap task (Elsabbagh et al., 2013), which measures the time taken to plan and execute a saccade from a centrally‐presented stimulus (CS), to a peripheral stimulus (PS) presented pseudo‐randomly at one side of the screen, at distance of ~20° of visual angle (at a viewing distance of 60 cm). Disengagement scores are the difference in RT to shift attention between two conditions, baseline (CS offset is timed to PS onset) and overlap (CS remains onscreen after PS onset); SM 1.3 for full details.

##### *Static visual attention* (Popout task; SM 1.3.3)

Infants were presented with a series of six annular visual arrays (10 s duration) each composed of five objects (face, car, bird, phone, scrambled face) in different locations on the screen (Gliga, Elsabbagh, Andravizou, & Johnson, 2009; Hendry et al., 2018). Gaze was averaged across eyes, assigned to an area of interest (AOI) and interpolated (<200 ms missing data). The key dependent variable is the proportion of gaze samples to the face divided by the total valid (non‐missing) gaze samples.

#### Measures of developmental outcome at 3‐years (SM 1.2)

Emerging ADHD traits (inattentive and hyperactive behaviour) were measured using the sum of raw scores from the ADHD sub‐scale of the Child Behaviour Checklist‐Preschool (CBCL‐P; Achenbach & Ruffle, 2000). ASD traits were measured with the Social Responsiveness Scale 2 Preschool Form (Constantino & Gruber, 2012); total raw scores are based on the sum of all items. Vineland Adaptive Behaviour Scales 2nd Edition (VABS; Sparrow, Cicchetti, & Balla, 2005) was used to measure adaptive Socialisation skills; standard scores are computed from developmental norms. Developmental level was measured with the Mullen Scales of Early Learning (MSEL) composite scores (Mullen, 1995; SM 1.2). Finally, experienced researchers supervised by a licenced clinical psychologist assigned a best estimate research diagnosis of DSM‐5 autism spectrum disorder (ASD+) or non‐autism (ASD−), informed by (but not dependent on) the ADOS‐2, ADI‐R and MSEL assessments and research observations at 2 and 3 years of age for every child, across all likelihood groups, that attended a 3 year visit (Table S2).

### Statistical approach

We reduced questionnaire measures of sleep with an initial factor analysis before using generalised estimating equations (GEEs) to examine likelihood group and outcome effects (see SM 2 for control analyses). Finally, we used structural equation modelling to look at the relation between sleep, social attention and later outcome phenotypes. We provide full details of this below.

Clinical questionnaire scores were excluded if >20% of items were missing (e.g., if parents had missed/been unable to answer more than 20% of questions for a subscale). Given the multiple sleep variables that could be extracted from the questionnaire data, we thought it pertinent to initially reduce the data. Factor analyses or similar analyses have previously been used successfully to group sleep questionnaire data in a variety of populations (Schoch, Huber, Kohler, & Kurth, 2020; Smith et al., 2008; Stores, Stores, Fellows, & Buckley, 1998). Moreover, the sleep variables extracted from the SSQ and the IBQ‐R were highly correlated with each other (Table S4; SM 3.2) and thus using a data reduction technique is appropriate to use in minimising the number of GEEs needed. In line with the expected relation between Day and Night Sleep, interpretation of the factors was clearer with an unrotated solution. The two‐factor solution is consistent with previous research that now examines the differential impact of Day and Night Sleep (as opposed to Total Sleep duration; Horváth & Plunkett, 2018; Lukowski & Milojevich, 2013). Indeed, previous research has also found that Night Sleep/Night Awakenings (as opposed to Day Sleep and Naps) is better able to differentiate between clinical and typical infant samples (Sadeh, 2004).

We conducted a factor analysis on the sleep measures across all three time points (Day Sleep duration, number of naps, Night Sleep Duration, Number of night awakenings and ISOP scores) using a Principal Components analysis with no rotation (in SPSS v25, IBM, NY, USA), resulting in two extracted factors (eigenvalues > 1) that were consistent with Day Sleep (23.2% variance) and Night Sleep (33.7% variance; see Tables S3 and S4 for factor loadings and correlations). We used individual factor scores for further analysis (though found comparable results when examining individual variables from our questionnaire measures; SM 2.3).

We then examined the effects of typical/elevated likelihood of ASD and/or ADHD and ASD outcome on sleep using separate generalised estimating equations (GEEs; compound symmetry, maximum likelihood) with the Day Sleep factor and the Night Sleep factor and the following fixed factors: Age (5‐months, 10‐months, 14‐months), Sex (male, female), Group (Likelihood/family history: ASD‐L, ADHD‐L and their interaction; or Outcome: ASD+, ASD−); and interaction terms of Group by Age. We also conducted sensitivity analyses when examining ASD Outcome, where we restricted the dataset to specific samples in the event of group differences inducing correlations. Of note, due to attrition in our sample (see Figure S1), there were fewer participants at the 3 year visit and thus within the ASD Outcome groups compared to the ASD likelihood groups.

We examined relations between sleep, social attention and later outcome phenotypes using cross‐lagged models estimated in an SEM framework (Mplus 7.13; Muthén & Muthén, 2000). Full Information Maximum Likelihood was used to account for missing data and Maximum Likelihood estimation was used to provide standard errors. A ‘good’ fit was indicated by RMSEA of 0.01–0.05, CFI of 1–0.95, and SRMR of <0.05 (Hu & Bentler, 1999; Kline, 2016). Coefficients were standardised with respect to the predictor and outcome (STDYX in Mplus). All models demonstrated a ‘good’ fit (Table S5). We also conducted a number of supplementary analyses controlling for age in days and eyetracking accuracy metrics (SM 2), where we found comparable results to those presented below.

## Results

We present the means and standard deviations of our sleep variables of interest (Day Sleep duration, Number of Day naps, Night Sleep duration, Night Awakenings and Infant Sleep Onset Problems) across our cohort in Table 2.

**Table 2:** Means (SD) of Day and Night Sleep variables that formed factor scores

	TL	EL	ASD−	ASD+
5‐months				
Day naps	2.71 (0.61)	3.2 (0.98)	3.13 (0.9)	2.85 (0.75)
Day Sleep duration (min)	137.5 (57.84)	129.7 (72.31)	131.04 (65.8)	124.5 (79.53)
Night awakenings	2.52 (1.48)	2 (1.49)	1.96 (1.45)	2.4 (1.91)
Night Sleep duration (min)	538.02 (161.87)	502.7 (184.35)	503.43 (179)	516 (157.99)
Average ISOP Scores	3.17 (1.38)	3.1 (1.5)	2.9 (1.3)	3.67 (2.19)
10‐months				
Day naps	2.05 (0.35)	2.15 (1.32)	2.17 (1.34)	1.95 (0.6)
Day Sleep duration (min)	127.02 (31.52)	124.15 (49.25)	120.48 (153.56)	143.5 (70.87)
Night awakenings	1.38 (1.08)	1.96 (1.55)	1.84 (1.53)	2.2 (1.87)
Night Sleep duration (min)	613.81 (125.59)	552.6 (153.28)	571.99 (153.56)	534 (50.6)
Average ISOP Scores	4.17 (1.05)	3.84 (1.21)	3.94 (1)	2.93 (1.24)
14‐months				
Day naps	1.63 (1.59)	1.57 (0.78)	1.56 (0.86)	1.6 (0.66)
Day Sleep duration (min)	140.79 (49.17)	116.39 (49.22)	119.24 (47.64)	118 (77.57)
Night awakenings	1.11 (1.51)	1.73 (1.48)	1.51 (1.38)	3.1 (1.58)
Night Sleep duration (min)	646.05 (104.7)	570 (131.57)	590.22 (136.1)	490.56 (147.06)
Average ISOP Scores	4.52 (0.84)	4.1 (1.26)	4.19 (1.15)	3.06 (1.44)

Higher ISOP scores indicate more sleep onset problems. Lower ISOP scores in our ASD+ group may be explained by greater variability due to the small sample size (*n* = 12). Sample demographics with ASD Outcome are only reported for the elevated likelihood (EL) groups. Elevated Likelihood includes all infants with a family history of ASD and/or ADHD (ASD‐L, ADHD‐L and ASD + ADHD‐L cohorts). EL‐ASD+ indicates participants who were ASD positive at the 3‐year diagnostic visit (ASD+ in the main text), whereas EL‐ASD− indicates participants who were ASD negative at this visit (ASD− in the main text). Please note ASD Outcome analyses in the main text also included TL infants.

### Effects of family history and ASD outcome

#### Day sleep

As hypothesised, Day Sleep scores decreased with Age [*F*(2, 204) = 13.4, *p* < .001, $\eta_{p}^{2}$ = .12] but did not differ by ASD‐L status [*F*(2, 204) = 2.46, *p* = .09, $\eta_{p}^{2}$ = .02]. There was no significant overall effects of either ASD‐L [*F*(1, 132) = 0.29, *p* = .59, $\eta_{p}^{2}$ = .002], ADHD‐L [*F*(1, 132) = 2, *p* = .16, $\eta_{p}^{2}$ = .01] or their interaction [*F*(1, 133) = 0.56, *p* = .73, $\eta_{p}^{2}$ = .004]. There was no effect of Sex [*F*(1, 135) = .21, *p* = .65, $\eta_{p}^{2}$ = .002] or significant interactions of Age*ADHD‐L; [*F*(2, 204) = 0.11, *p* = .89, $\eta_{p}^{2}$ = .001] or ASD*ADHD‐L*Age [*F*(2, 204) = 1.54, *p* = .22, $\eta_{p}^{2}$ = .01].

In the model including ASD Outcome, Day Sleep scores decreased with Age [*F*(1, 168) = 3.46, *p* = .03, $\eta_{p}^{2}$ = .02; $\eta_{p}^{2}$ = .04 when restricting analyses to our ASD‐L sample], with a significant though weak interaction between ASD Outcome and Age such that the ASD+ group showed a shallower decrease in Day Sleep scores [*F*(2, 168) = 3.43, *p* = .04, $\eta_{p}^{2}$ = .04; $\eta_{p}^{2}$ = .04 in the restricted sample analyses]. Post hoc *t*‐tests showed no difference between groups at any specific time point [5 months: *t*(72) = 1.02, *p* = .16, *d* = 0.35; 10 months: *t*(98) = 1.57, *p* = .12, *d* = 0.53; 14 months: *t*(96) = 0.41, *p* = .68, *d* = 0.14].

#### Night sleep

**Figure 1:**
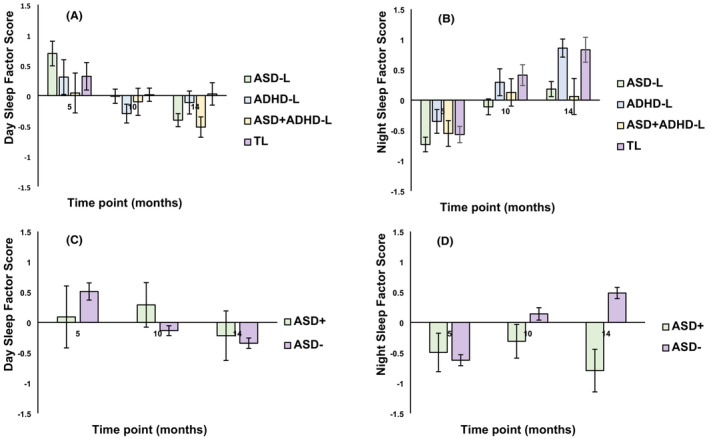
Graphs showing mean Sleep factor scores across elevated and typical likelihood groups (Day Sleep: Panel A; Night Sleep: Panel B) and ASD Outcome groups (Day Sleep: Panel C; Night Sleep: Panel D) [Color figure can be viewed at wileyonlinelibrary.com]

As expected, Night Sleep scores increased with age [*F*(2, 199) = 43.78, *p* < .001, $\eta_{p}^{2}$ = .31]. However, infants with an elevated likelihood of ASD had lower Night Sleep scores than those without an elevated likelihood of ASD [*F*(1, 133) = 10.08, *p* = .002, $\eta_{p}^{2}$ = .07] (Figure 1). There was no significant effect of ADHD‐L [*F*(1, 133) = 0.17, *p* = .68, $\eta_{p}^{2}$ = .001] or ASD*ADHD‐L [*F*(1, 133) = 0.03, *p* = .87, $\eta_{p}^{2}$ = .0], nor any interactions between ASD‐L, ADHD‐L and Age, ASD‐L*Age [*F*(2, 199) = 1.77, *p* = .17, $\eta_{p}^{2}$ = .02], ADHD‐L*Age [*F*(2, 199) = 0.76, *p* = .47, $\eta_{p}^{2}$ = .008], ASD*ADHD‐L*Age [*F*(2, 199) = 0.65, *p* = .53, $\eta_{p}^{2}$ = .006]. There was no significant effect of Sex [*F*(1, 135) = 1.01, *p* = .32, $\eta_{p}^{2}$ = .007].

In the model including Outcome, Night Sleep scores decreased from 5‐ to 10‐months then increased from 10‐ to 14‐months [main effect of Age, *F*(2, 166) = 5.42, *p* = .005, $\eta_{p}^{2}$ = .06; $\eta_{p}^{2}$ = .04 when restricting analyses to our ASD‐L sample]; overall, the ASD+ group had lower Night Sleep scores than the ASD‐ group [*F*(1, 109) = 4.6, *p* = .03, $\eta_{p}^{2}$ = .04; $\eta_{p}^{2}$ = .03 in the restricted sample analyses]. Effects of Age varied with ASD Outcome [*F*(2, 166) = 9.68, *p* < .001, $\eta_{p}^{2}$ = .1; $\eta_{p}^{2}$ = .08 in restricted sample analysis]; the ASD+ group had lower Night Sleep scores than the ASD‐ group at 14‐months [*t*(96) = 4.1, *p* < .001, *d* = 1.46; *d* = 0.47 with restricted sample], but not at 5‐ [*t*(72) = .49, *p* = .62, *d* = 0.16] or 10‐months [*t*(98) = 1.41, *p* = .16, *d* = 0.47] (Table 2, Figure 1).

Given the weak or non‐significant effects of ASD likelihood on our Day Sleep factor scores, hereafter we focus only on Night Sleep.

To assess whether the relation to later ASD could be affected by the presence of ADHD traits, [given their association: *r*(100) = .56, *p* < .001], we examined how Night Sleep at 14‐months relates to later (3‐year) dimensional measures of autism and ADHD, using partial parametric correlations. When partialling out CBCL scores, we found that greater Night Sleep associated with lower autistic traits [SRS; *r*(85) = −.25, *p* = .02]. However, there was no association with ADHD traits when controlling for SRS scores [CBCL; *r*(85) = −.04, *p* = .75; Figure S2]. Thus, this suggests that the relation between infant sleep and autistic traits is more direct.

### Sleep and visual attention

#### Attention shifting: Disengagement (Gap‐overlap task)

Night Sleep scores were stable from 5 to 10‐months and 10 to 14‐months (*β* = .33, *p* = .002 and *β* = .72, *p* < .001). Disengagement scores showed longitudinal stability from 5 to 10‐months and 10 to 14‐months (*β* = .25, *p* = .03 and *β* = .43, *p* < .001). There were no significant cross‐lagged effects from Night Sleep at one timepoint to visual attention at another or vice versa (*p*s > .2; Table S5), nor were there any concurrent relationships between Sleep and Disengagement at any time point (*p*s > .2; Table S5).

#### Social attention in static stimuli (Popout task)

**Figure 2:**
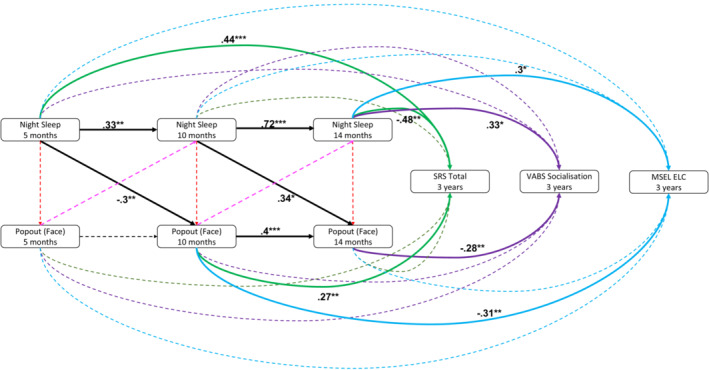
Associations between Night Sleep, Looking to the Face during the Static social attention (Popout) task and dimensional outcomes. Dashed lines represent non‐significant associations, solid lines indicate significant associations (*p < .05, **p < .01, ***p < .001) [Color figure can be viewed at wileyonlinelibrary.com]

Mean proportion of Face Looking was stable between 10‐ and 14‐months (*β* = .41, *p* < .001), but not 5‐ to 10‐months (*β* = .14, *p* = .22). Lower Night Sleep scores at 5‐months associated with increased mean proportion of Face Looking at 10‐months (*β* = −.3, *p* = .005). In comparison, higher Night Sleep scores at 10‐months associated with higher mean proportion of Face Looking at 14‐months (*β* = .34, *p* = .02). We found no concurrent relationships between Sleep and Face Looking at any time point or cross‐lagged associations from Face Looking to Sleep (Figure 2; Table S5).

#### Sleep, attention and later traits

Given the interrelations between sleep and visual attention to static stimuli, we then tested their joint relations to later cognitive, adaptive and autistic trait outcomes in separate structural equation models (summarised in Figure 2). In the model including SRS, higher SRS scores at 3 years associated with increased Night Sleep scores at 5‐months and decreased Night Sleep scores at 14‐months (*β* = .44, *p* < .001 and *β* = −.48, *p* = .001 respectively). Further we found that increased Face Looking at 10‐months associated with increased SRS scores at 3‐years (*β* = .26, *p* = .02). We found no other significant associations (all *p*s > .12, Table S5).

In the model including the Vineland Socialisation scale, higher Night Sleep scores at 14‐months was related to higher Socialisation scores at 3‐years (*β* = .34, *p* = .03). Increased Face Looking at 14‐months was related to lower Socialisation scores at 3‐years (*β* = −.28, *p* = .01). We found no other significant associations (all *p*s > .1, Table S5).

In the model including the MSEL Early Learning Composite, lower Face Looking at 10‐months was related to higher ELC scores at 3‐years (*β* = −.3, *p* = .01). Finally, higher Night Sleep scores at 14‐months related to higher ELC scores at 3‐years (*β* = .3, *p* = .03; Table S5). We found no other significant associations (all *p*s > .12, Table S5).

## Discussion

Disruptions in sleep are one of the most common associated symptoms of neurodevelopmental conditions like autism and ADHD (Cortese et al., 2009, 2020) and individual differences in early sleep have been linked to infant attention (Bernier et al., 2013; Sadeh et al., 2015) and later cognitive and social functioning in the general population (Tham et al., 2017; Tomisaki et al., 2018). Thus, in the present study we examined the prospective relation between sleep, attention, and later cognitive and social functioning in a cohort enriched for ASD and ADHD outcome. We show that in the first 2 years of life, infants with a family history of ASD demonstrate reduced sleep factor scores (as indexed by shorter sleep durations, increased night waking and more difficult settling). By 14 months of age, reduced sleep factor scores related to ASD outcome at age 3 years. Individual differences in infant night sleep scores were also dimensionally related to later social functioning, cognitive ability and dimensional ASD symptomatology; and there were prospective associations between night sleep and visual attention from infancy. Although there were no effects of family history on day sleep scores, infants with later ASD showed a slower decrease in day sleep with age. Together, our results suggest that sleep difficulties may associate with later autism and related developmental difficulties from the beginning of the second year of life.

### Sleep and clinical outcomes

Whilst there is much research examining childhood sleep problems in neurodevelopmental disorders such as ASD and ADHD, there is comparatively little literature in terms of the utility of sleep as a prognostic marker for these disorders. Here, we examined combined measures of sleep duration, interruptions and nap frequency and sleep onset problems. Altered night sleep was observed across timepoints in infants with a family history of ASD, and by 14‐months individual differences related to both later autism outcome, autistic traits, cognitive functioning and social adaptive skills. Of note, 14 months is often associated with the first clear emergence of behavioural autism symptoms in some children (Tanner & Dounavi, 2021), and it may be that consolidated sleep problems emerge on the same timetable; however, there were no concurrent associations between night sleep and an infant measure of autism traits (SM 2). Our findings are consistent with a broad literature showing concurrent links between poor night sleep and cognition, social functioning and autism in older children (Shaw et al., 2022; Veatch et al., 2017); and prospective general population studies showing links between infant sleep and later cognitive and social functioning (Tomisaki et al., 2018).

Our analyses used a data‐driven approach to elicit a Night Sleep Factor and analyses by specific sleep parameters (such as duration, onset, awakenings, total sleep or sleep efficiency) produced weaker effects (SM 2). Indeed, in the previous literature different studies find associations between different sleep features and developmental status (e.g. increased night awakenings and lower cognitive scores; Scher, 2005; longer sleep durations and increased social and cognitive abilities; Tomisaki et al., 2018, sleep onset problems and later ASD; MacDuffie et al., 2020). It may be that the overall quality of night sleep is best captured by joint analysis of multiple sleep features in the second year of life, particularly when using questionnaire measures.

Sleep difficulties were clearly related to multiple aspects of developmental outcome by the second year of life. However, the pattern was different in the first year: whilst at 14 months *higher* Night Sleep scores were associated with *fewer* ASD traits, *higher* Night Sleep scores at 5‐months were associated with *greater* ASD traits at 3‐years (SRS). It is unlikely that this effect solely represents change in the meaning of the Night Sleep Factor with time since the same broad pattern is seen using the individual constituent measures that comprise the Night Sleep Factor (SM 2.2) and with a new version of the factor scores omitting the sleep onset problem score, the only variable that showed evidence of differences in factor loading over the three timepoints (SM 2.3). Changes in parental reporting may be relevant; parent‐reported sleep systematically varies in its relation to actigraphy‐captured data as a function of maternal stress, individual differences, and infant age (Gossé, Wiesemann, Elwell, & Jones, 2022).

Beyond measurement properties, the observed pattern could also represent a reduced rate of developmental change, with greater change in sleep patterns over time associating with fewer ASD traits. Indeed, slower change in day sleep was related to later autism outcome; further work should examine whether this is linked to direct changes in learning and memory pre‐post sleep (Lukowski & Milojevich, 2013). Alternatively, there may be a subgroup of infants who show high levels of passivity and long sleep durations (temperamentally ‘easy babies’) in early infancy who later develop symptoms of ASD (potentially reflected in lower surgency that increases by 24 months; Paterson et al., 2019). The sleep onset problems that related to later autism from 6 months in previous work (MacDuffie et al., 2020) may represent an earlier‐emerging indicator of developmental differences than the broader profile of night sleep or may reflect broader increases in temperamental negativity linked to difficulty in settling (Pijl et al., 2019), indeed, sleep onset problems loaded differently on the Night and Day factors with age (see SM 2). As such, it is important to consider the age‐related context in future research when developing sleep screeners or sleep interventions; for example, actively increasing sleep durations in the first year of life may not necessarily be beneficial to later outcomes.

We found no differences in sleep in relation to family history of ADHD. Further, whilst increased Night Sleep scores were related to lower ADHD CBCL scores, this relationship become non‐significant when controlling for ASD symptoms. These findings contrast with prior research in newborns with a family history of ADHD that demonstrated increased variability and decreased stability in measures of day sleep (Landau et al., 2010) and predictive relations between broad measures of infant regulatory problems and later ADHD (Bilgin et al., 2020; Hemmi, Wolke, & Schneider, 2011). Future work should examine the potential confounding role of cognitive ability or ASD traits in studies focusing on ADHD. It is also possible that childhood follow‐up will identify relations between sleep and ADHD in our cohort, given diagnoses of ADHD are not typically made until mid‐childhood or later (Bélanger, Andrews, Gray, & Korczak, 2018).

### Sleep and visual attention

Consistent with previous evidence of links between sleep and attention control (O'Callaghan et al., 2010), we identified developmental interrelations between sleep and social attention that were not observed in our non‐social attention‐shifting task. Notably, we observed predictive, but not concurrent, associations from earlier sleep to later visual attention and did not observe relations from earlier visual attention to later sleep. This is consistent with a potential causal effect of poor infant sleep on the developing attention system, rather than a concurrent associative effect such that infants who are more tired on the testing day show poorer attention. Matching the relation between sleep and ASD Outcome, we observe developmental changes in the direction of the relationship between sleep and visual attention across the first years of life. H*igher* Night Sleep scores at 10‐months were related to *more* Looking at the Face at 14‐months, which associated with *lower* Socialisation skills at 3 years. In this simple task with static stimuli, more looking is less optimal for older infants, who should be strategically scanning the array rather than dwelling on the most salient face stimulus (Elsabbagh et al., 2013). In this task, we have previously shown that a failure to decrease attention to the face between 10‐ and 14‐months relates to poorer later executive functioning skills (Hendry et al., 2018) and longer durations of looking at 14‐months are seen in infants with vs without a family history of ASD (Gui et al., 2020). Taken together, this may suggest that more consolidated night sleep in younger infants is not necessarily beneficial for visual attention; for example, it may be that infants whose caregiver notices more awakenings during the night are receiving increased opportunities for social input during an accelerated period of brain development. Of note, earlier in development, *lower* Night Sleep scores at 5‐months were related to *more* looking at the Face at 10‐months, which again associated with *poorer* cognitive development and *more* ASD traits at 3‐years. However, this was not apparent in an analysis of Night Factor scores omitting the sleep onset problems score (SM 2) and should therefore be treated with caution.

## Summary and implications

Sleep may be a viable target for early intervention in infants with a family history of ASD, particularly around the age of 14‐months. At this age, Table 2 shows that infants with a family history of ASD were sleeping on average 70 min less per night than infants without a family history of ASD. Further, infants with later ASD were sleeping on average 100 min less per night than infants with other developmental outcomes and woke up twice as much. This degree of sleep disruption is likely to not only cause difficulties for the baby, but to have a significant impact on the family. Sleep is malleable (especially in the first 2 years of life; Mindell et al., 2011), with many studies reporting decreased sleep onset latency, night awakenings and increased night sleep duration with intervention (Reuter, Silfverdal, Lindblom, & Hjern, 2020). As such, interventions targeting sleep disturbances may be a particularly fruitful avenue of research. In comparison to social attention interventions (which are often resource intensive in terms of funding, time and accessibility), sleep intervention programmes may offer a more scalable and accessible option for parents from a variety of socio‐economic and family backgrounds, as they can be performed in participants' home and initial training can even be delivered virtually (Mindell et al., 2011). Earlier in infancy, measures of visual attention could provide early readouts of the efficacy of sleep intervention, but the heterogenous direction of effects indicates that further work is required to determine the nature of optimal sleep for the infant brain.

### Limitations and future directions

Due to the sample size, we did not examine familial history and ASD outcome within our longitudinal association models. Despite this, we observed evidence of changes in sleep associating with measures that also change with ASD (e.g., looking to social stimuli, socialisation behaviours and ASD traits). The current study focused on sleep in the first 14‐months; further work should look beyond infancy (which is often a time of dramatic changes in sleep) and examine sleep in toddlerhood, when it becomes even more stable. Examining sleep at 3 years of age would reveal concurrent associations between sleep and ASD symptomatology. We used parent‐report measures of sleep, which can be affected by factors like SES or mental health (Herbers, Garcia, & Obradović, 2017); integration of objective measures like actigraphy will be an important future step.

Further to this, capturing comparable measures of infant sleep over developmental time is difficult and is the topic of active investigation (Schoch et al., 2020; Tikotzky & Volkovich, 2019). Our factor analysis approach showed a similar underlying sleep structure across multiple variables (Night Awakening, Night Duration and Day Naps) from 5 to 14 months and produced factors with good developmental stability. However, one of our key variables (ISOP; infant sleep onset problems which captures an infant's ability to settle to sleep and which has been used in previous work in this area and thus was essential to reproducibility; MacDuffie et al., 2020) was related differently to other aspects of night sleep at 5 months relative to older timepoints. Parental settling to sleep practices change over the first years of life, with many infants transitioning from co‐sleeping, being rocked or fed to sleep to sleeping in their own cot or room; the timing of this transition is likely not independent of the infant's ability to sleep through the night. This may lead to different relationships between settling to sleep and infant sleep quality at different developmental stages. In the present study, the fact that the 5 month sleep factor was slightly less coherent should be considered when examining age‐related change in the relation between sleep and outcome measures. Though it is important to note that the reversal in association between Night Sleep and SRS scores between 5 and 14 months was also present when the Sleep Onset Problems variable was removed from the model.

### Summary

We show that infants with a family history of ASD, and those with an ASD diagnosis at 3‐years, slept less at night by 14‐months (as indexed by shorter night sleep durations, increased frequency of night awakenings and increased sleep onset problems). This association was not found for those with a family history of ADHD. Further, lower Night Sleep scores at 14‐months was related to weaker cognitive skills, social adaptive functioning and greater ASD traits in toddlerhood. Sleep in early infancy predicted changes in social attention that related to subsequent cognitive and social outcomes. Our results suggest the importance of exploring infant sleep interventions for populations with a family history of ASD.

## Supporting information


**SM 1.** Methods.
**SM 2.** Results.
**SM 3.** Table and figures.
**SM 4.** References.
**SM 5.** Appendix.
**Figure S1.** Consort diagram of number of participants for each measure at each time point.
**Figure S2.** Scatter graphs showing Night Sleep Factor.
**Table S1.** Categorisation of elevated likelihood cohorts.
**Table S2.** Means and SE for MSEL across Group.
**Table S3.** Table showing factor loadings for each sleep variable from the SSQ and IBQ‐R.
**Table S4.** Table showing correlations between individual variables.
**Table S5.** Table showing beta coefficient, *p* values and fit statistics for crossed lagged models.
