# Supplementary material for: Infant sleep predicts trajectories of social attention and later autism traits
Source: J Child Psychol Psychiatry. 2023 Mar 29;64(8):1200–11. doi: 10.1111/jcpp.13791 (PMC10952761; doi:10.1111/jcpp.13791)
Supplement: Supplementary file 1 — SM 1. Methods. SM 2. Results. SM 3. Table and figures. SM 4. References. SM 5. Appendix. Figure S1. Consort diagram of number of participants for each measure at each time point. Figure S2. Scatter graphs showing Night Sleep Factor. Table S1. Categorisation of elevated likelihood cohorts. Table S2. Means and SE for MSEL across Group. Table S3. Table showing factor loadings for each sleep variable from the SSQ and IBQ‐R. Table S4. Table showing correlations between individual variables. Table S5. Table showing beta coefficient, p values and fit statistics for crossed lagged models. [file JCPP-64-1200-s001.docx]

TITLE: INFANT SLEEP PREDICTS LATER ASD TRAITS AND TRAJECTORIES OF SOCIAL ATTENTION: Supplementary Materials

Jannath Begum-Ali^1*^, Louisa K. Gossé^1*^, Luke Mason^1^, Greg Pasco^2^, Tony Charman^2^, Mark H. Johnson^1,3^, Emily J.H. Jones^1^ and the STAARS Team^φ^

Table of Contents

[1 Methods 4](#_Toc126145688)

[1.1 Further information on cohort ascertainment 4](#_Toc126145689)

[1.2 Further behavioural measurement information 5](#_Toc126145690)

[1.2.1 Sleep and Settle Questionnaire (SSQ) 5](#_Toc126145691)

[1.2.2 Infant Behaviour Questionnaire Revised (IBQ-R) 5](#_Toc126145692)

[1.2.3 Child Behaviour Checklist-Preschool (CBCL-P) 6](#_Toc126145693)

[1.2.4 Autism Diagnostic Schedule Toddler Module Second Edition (ADOS-2) 6](#_Toc126145695)

[1.2.5 Autism Diagnostic Interview Revised (ADI-R) 6](#_Toc126145696)

[1.2.6 Social Responsiveness Scale 2 Preschool Form (SRS) 6](#_Toc126145697)

[1.2.7 Vineland Adaptive Behaviour Scales (VABS) 6](#_Toc126145698)

[1.2.8 Mullen Scales of Early Learning (MSEL) 6](#_Toc126145699)

[1.3 Further details of eyetracking measures 9](#_Toc126145700)

[1.3.1. Gap-overlap task 9](#_Toc126145701)

[1.3.1.1. Stimulus presentation 9](#_Toc126145702)

[1.3.1.1 Data extraction: 10](#_Toc126145703)

[1.3.2 Popout task 10](#_Toc126145704)

[1.3.2.1 Stimulus presentation: 10](#_Toc126145705)

[1.3.2.2 Data extraction: 10](#_Toc126145706)

[2. Results 12](#_Toc126145707)

[2.1 Analysis 1: Controlling for eye tracking accuracy 12](#_Toc126145708)

[2.2 Analysis 2: Individual variables 12](#_Toc126145709)

[2.3 Analysis 3: Confirmatory Factor Analysis 13](#_Toc126145710)

[2.4 Analysis 4: Total Sleep Duration 14](#_Toc126145711)

[2.5 Analysis 5: Concurrent relationships between Night Sleep and ASD symptoms 14](#_Toc126145712)

[2.6 Analysis 6: ISOP score control analyses 14](#_Toc126145713)

[3. Tables and figures referred to in the main text 16](#_Toc126145714)

[3.1 PCA factor loadings for Day and Night Sleep 16](#_Toc126145715)

[3.2 Bivariate correlations between individual sleep variables 16](#_Toc126145716)

[3.3. Cross lagged models between Sleep, Visual Attention and later traits 17](#_Toc126145717)

[3.4 Scatter plots of Night Sleep and later traits 19](#_Toc126145718)

[4. References 20](#_Toc126145719)

[5. Appendix 22](#_Toc126145720)

# 1 Methods

## 1.1 Further information on cohort ascertainment

Participants were recruited for a longitudinal study running from 2013 to 2019 from a volunteer database, community flyers, internet adverts and clinical networks. Participant families were reimbursed expenses for travel, subsistence and overnight stay if required. Infants were given a certificate and t-shirt after each visit.

Information about diagnostic status was ascertained through a number of methods. Before families enrolled in the study, a telephone screening form was used to determine the presence of ASD and ADHD in family members. During their infant’s visit to the lab, the parent/caregiver also completed a “Medical and Psychiatric History Interview” (SM5: Appendix A) with the researcher. The telephone screening form and this formal interview at a study visit were the primary sources of information about diagnostic status. In addition, we asked for medical updates at each study visit and re-administered the Medical and Psychiatric History Interview at the 2-year timepoint. We also requested diagnostic letters and asked parents to complete the DAWBA (Goodman, Ford, Richards, Gatward & Meltzer, 2000) ASD and ADHD sections and these were reviewed by the senior clinician (TC). In addition, parents completed the Conners (Conners, 2008) (for ADHD) and the Social Communication Questionnaire (Rutter, Bailey & Lord, 2003) and Social Responsiveness Scale (Constantino, 2012; for ASD) on the family member with a diagnosis and where possible all other family members. This information is used to characterise our sample rather than for exclusionary purposes since, in the UK, NHS clinical diagnoses follow a gold-standard procedure including collation of information from parents, teachers and from in-person assessment that is beyond the scope of this study and more accurate than simple questionnaire measures.

Up to 30% of children with ASD meet criteria for ADHD when prospectively assessed (Simonoff, Pickles, Charman, Chandler, Loucas & Baird, 2008). In clinical practice, the prevalence of dual diagnosis is in practice much lower (Russell, Rodgers, Ukoumunne & Ford, 2014). Given the nature of the co-occurrence between ASD and ADHD and our longitudinal study, sometimes family members would have a suspected diagnosis of ADHD at study entry that would be confirmed later in the study; on other occasions, a family would enrol on the basis of an ASD diagnosis in an older sibling but by the end of the study, they would report that the same sibling was now undergoing assessment for suspected additional ADHD. Where possible, families who reported suspected ADHD at study entry were screened using a shortened version of the Conners. For siblings (aged less than 6 years), a shortened version of the Conners Early Childhood (Conners, 2008, Conners & Goldstein, 2009) form is used. For siblings (6 years or older), a shortened version of the Conners 3 was used. Thresholds for inclusion were the presence of 6 ADHD traits on either the hyperactivity/impulsivity or inattention scale, and a positive score on the impairment scale. For parents a shortened version of the Conners Adults ADHD Rating Scale (CAARS) was used. Thresholds for inclusion were the presence of 5 ADHD traits on either the hyperactivity/impulsivity or inattention scale as per updated DSM V guidelines (see Table 1 for categorisation of the cohort). In terms of the use impairment scores, we used a reduced version of the Conners EC and Conners 3 for individuals under 18 and the CAARS for individuals aged 18+ years. The Conners EC and Conners 3 included questions regarding impairment, as such we also included these questions in our screening forms. In comparison, the CAARS (adult questionnaire) did not include questions regarding impairment. In order to maintain consistency of measure, we did not adapt the CAARS to add impairment questions. Of note, at initial contact with participants, parents were asked if there were any diagnoses of ADHD in the immediate family or if they had any concerns about ADHD. It is only if parents reported concerns that the screening process took place. This is a very similar categorisation protocol to that adopted by other papers/labs using the prospective longitudinal study model in infants at elevated likelihood of ADHD (see Miller et al., 2020; see Table S1 for the number of screened vs diagnosed participants). Families who screened positive on this instrument were then included as a confirmed case. However, it remains likely that within families with ASD, rates of actual ADHD are higher than those captured by our 1/0 diagnostically-based rating system.

| Table S1: Categorisation of elevated likelihood cohorts | | | |
| --- | --- | --- | --- |
|  | **ASD-L** | **ADHD-L** | **ASD+ADHD-L** |
| Parent reported diagnosis in older sibling | 74 | 7 (+3^) | 15 |
| Parent reported diagnosis in parent | 3 | 18 | 2 |
| Parent reported diagnosis in both older sibling+parent | 3 | 1 | 2 |
| Screened parent (for ADHD traits) |  | 1 | 0 (+1)* |
| Screened older sibling (for ADHD traits) |  | 4 | 0 (+1)** |

*Sibling diagnosed with ASD, also screened for ADHD traits . **Sibling diagnosed with ASD, parent screened for ADHD traits. ^3 participants with half siblings diagnosed with ADHD; these participants were only included in Analysis 2: ASD Outcome

## 1.2 Further behavioural measurement information

### 1.2.1 Sleep and Settle Questionnaire (SSQ)

The SSQ is a 34-item parent report questionnaire that assesses infant sleep and settling behaviour. We examined the 6 questions relating to how long infants sleep during both the day and the night, as well as the number of naps taken and the number of awakenings during the night. For items relating to sleep durations, responses were transformed to minutes (as parents often responded with a mix of hours and minutes across the cohort e.g., ‘7 hours’ was transformed to ‘420 minutes’). Of note, it is unlikely to be possible from parent report measures to get minute level data for durations. If parents reported a range (e.g., 1-2 naps), the mean value was taken (1.5 naps). Participants were excluded from specific Day/Night analyses if they had missing data for that analysis.

### 1.2.2 Infant Behaviour Questionnaire Revised (IBQ-R)

The short form version of the IBQ-R includes three of the ISOP (Infant Sleep Onset Problems) questions rated on a scale from never (1) to always (7); specifically “How often did your baby have a hard time settling down to sleep?”, “How often did your baby fall asleep within 10 minutes?” and “How often did your baby settle down to sleep easily?” (with the latter two items reverse scored). From these questions, we extracted the ‘Infant sleep onset problems’ variable (ISOP; MacDuffie et al., 2020).

### 1.2.3 Child Behaviour Checklist-Preschool (CBCL-P)

### Parents are asked to indicate how well each statement describes their child’s behaviour as observed within the past 2 months on a 3-point Likert rating. Questions for the ADHD subscale included items such as: “My child can’t concentrate/pay attention for too long” and “My child can’t sit still/is restless/hyperactive”. Parents rated their child from (0) not true at all to (2) Very true/often true. We summed the 6 questions from the ADHD subscale to get a total CBCL ADHD subscale score.

### 1.2.4 Autism Diagnostic Schedule Toddler Module Second Edition (ADOS-2)

The ADOS-2 (Lord et al., 2012) is considered the “gold standard” instrument in the diagnostic process for ASD. The ADOS-2 is a semi-structured play-based assessment that evaluates the presence of ASD symptoms across the domains of social communication, restricted interests and repetitive behaviours. The measure was administered by trained researchers in the STAARS team. All assessments were consensus scored by a second trained researcher on the day of administration.

### 1.2.5 Autism Diagnostic Interview Revised (ADI-R)

The ADI-R (Lord et al., 1994) is a semi-structured parent interview conducted by a trained researcher. Parents are asked about their child in relation to language and communication abilities, reciprocal social interactions and restricted, repetitive and stereotyped behaviours and interests. The ADI-R was carried out at the 3 year time point only.

### 1.2.6 Social Responsiveness Scale 2 Preschool Form (SRS)

ASD traits were measured with the Social Responsiveness Scale 2 Preschool Form (Constantino & Gruber, 2012). Parents are asked to indicate how well each statement describes their child’s behaviour on 4-point Likert rating (e.g., 1 = ‘Not true’, 2 = ‘sometimes true’, 3 = ‘often true’, 4 = ‘almost always true’); total t-scores are based on the sum of all items relative to sex-specific norms.

### 1.2.7 Vineland Adaptive Behaviour Scales (VABS)

Vineland Adaptive Behaviour Scales (Sparrow et al., 2005) was used to measure adaptive Socialisation skills. Parents are asked to indicate how well each statement describes their child’s abilities on a 3-point Likert rating (e.g., 0 = ‘Not yet’, 1 = ‘partially’, 2 = ‘yes’) and standard scores are computed from developmental norms.

### 1.2.8 Mullen Scales of Early Learning (MSEL)

The MSEL (Mullen, 1995) is a standardised measure that assesses developmental ability across five domains: Gross Motor, Visual Reception, Fine Motor, Receptive Language and Expressive Language. These five domains are then used to compute the MSEL Early Learning Composite (ELC) standard score. The MSEL was administered trained researchers in the STAARS team. To allow for the greatest level of replicability and consistency across examiners, we have extremely strict guidelines about how Mullens should be administered and marked. To this end, our guidelines for Mullen scoring include only behaviours that are captured on camera (so can be confirmed by a second/third researcher if necessary) within the MSEL session. For example, if an infant demonstrates babbling throughout the rest of the testing day (i.e., during another task or a lunch break), but not during the specific MSEL administration session, we would not score this infant as being able to produce babbling sounds on the Expressive Language scale. To further ensure the fidelity of the scoring, a second fully trained researcher watches the administration in real time (via a video feed) and consensus discussions take place after the testing session. These strict administration and scoring guidelines (although those recommended in the Mullen manual) may not be those applied more broadly in the field, and thus may account for relatively poorer performance in this cohort at infant timepoints relative to US norms (Jones, Mason et al., 2019). We present standard scores for overall developmental level for descriptive cohort comparisons (see Table S2).

| Table S2: Means and SE for MSEL across Group | | | | | | |
| --- | --- | --- | --- | --- | --- | --- |
|  | Fine motor | Gross motor | Visual reception | Receptive Language | Expressive Language | Early Learning Composite |
| 5 months |  |  |  |  |  |  |
| TL | 42.92 (2.05) | 43.69 (1.85) | 47.27 (1.24) | 36.88 (2.21) | 41.85 (1.46) | 85 (1.83) |
| ASD-L | 43.02 (1.34) | 47.08 (1.09) | 46.08 (1.35) | 34.67 (1.72) | 40.65 (1.05) | 82.96 (1.51) |
| ADHD-L | 44.56 (1.57) | 48.56 (1.7) | 44.56 (1.97) | 41.19 (3.51) | 37.69 (2.75) | 84.5 (2.45) |
| ASD+ADHD-L | 42.92 (2.27) | 49 (1.17) | 48.25 (2.52) | 43.42 (4.21) | 40.5 (2.15) | 87.75 (2.8) |
| 10 months |  |  |  |  |  |  |
| TL | 51.63 (2.48) | 34.89 (2.27) | 48.85 (1.54) | 39.26 (1.72) | 36.85 (1.9) | 88.89 (2.35) |
| ASD-L | 50.24 (1.34) | 38.25 (1.11) | 49.82 (1.08) | 38 (1.21) | 36.53 (1.47) | 88.03 (1.73) |
| ADHD-L | 51.85 (2.64) | 38.3 (2.05) | 47.26 (1.86) | 34.67 (1.96) | 33.85 (2.34) | 84.67 (2.97) |
| ASD+ADHD-L | 49.21 (2.93) | 36.16 (2.2) | 47.68 (1.77) | 35.63 (2.47) | 36.58 (3.42) | 85.42 (3.86) |
| 14 months |  |  |  |  |  |  |
| TL | 49.65 (2.54) | 36.74 (2.79) | 35.09 (1.85) | 32.87 (1.35) | 37.09 (1.83) | 78.78 (2.5) |
| ASD-L | 48.22 (1.36) | 46.64 (1.55) | 37.67 (1) | 31.48 (1) | 36.67 (1.33) | 78.37 (1.39) |
| ADHD-L | 47.21 (2.51) | 45.5 (2.21) | 36.33 (1.19) | 31.54 (1.88) | 40.75 (1.91) | 79.08 (2.27) |
| ASD+ADHD-L | 43.89 (2.27) | 45.58 (2.64) | 33.47 (1.49) | 28.52 (2.45) | 34.37 (3.15) | 72.53 (3.33) |
| 2 years |  |  |  |  |  |  |
| TL | 55.31 (2.49) |  | 59.63 (2.18) | 57.67 (1.78) | 55.42 (2.51) | 114.25 (3.66) |
| ASD-L | 50.81 (1.32) |  | 48.89 (1.62) | 51.06 (1.73) | 49.74 (1.86) | 100.63 (2.62) |
| ADHD-L | 51.31 (2.28) |  | 56.23 (2.55) | 52 (2.95) | 52.59 (2.93) | 106.86 (4.52) |
| ASD+ADHD-L | 51.37 (2.76) |  | 47.94 (2.57) | 49 (2.6) | 44.94 (2.77) | 96.94 (4.28) |
| 3 years |  |  |  |  |  |  |
| TL | 64.68 (2.6) |  | 67.58 (2.14) | 65.84 (1.45) | 60.84 (1.95) | 129.05 (2.7) |
| ASD-L | 50.59 (2.04) |  | 58.7 (1.74) | 54.25 (1.74) | 51.42 (1.51) | 108.1 (2.39) |
| ADHD-L | 58.09 (3.28) |  | 63.39 (3) | 56.7 (2.83) | 53.78 (2.52) | 118.39 (1.93) |
| ASD+ADHD-L | 51.38 (3.94) |  | 58.31 (4.15) | 52.2 (3.29) | 44.2 (3.21) | - 1. 5.14) |

## 1.3 Further details of eyetracking measures

1.3.1. Eye tracking tasks were administered as part of a larger battery (~30 minutes), using a Tobii TX-300 eye tracker (Tobii AB, Stockholm, Sweden) sampling at 120 Hz. The screen had a diagonal size of 23” (58.4 cm ×28.6 cm, 52.0◦×26.8◦@ 60 cm), a native resolution of 1920 ×1080 pixels and an aspect ratio of 16:9. Stimuli were presented on Apple (Apple Inc., Cupertino, CA, USA) Macbook Pro computers, using our custom-written stimulus presentation framework (Task Engine, sites.google.com/site/taskenginedoc/), running in Matlab R2020b (The MathWorks Inc., Natick, MA, USA) using Psychtoolbox 3.

Before calibration started, participants viewed an infant-friendly video. The experimenters saw the gaze data visualised, and were guided as to positioning the participant relative to the eye tracker. Separate visual indicators showed the distance from the participant’s current position to the centre of the tracker box (where data quality is highest), in three dimensions. Once the experimenter was satisfied that the participant’s eyes were being tracked and their positioning was optimal, they moved to the calibration phase. Here a five-point calibration was automatically run, using infant-friendly stimuli (a colourful spiral which rotated as it shrank to engage attention). If enough valid data was collected after five calibration points, the results were plotted and presented to the experimenter, otherwise the five-point procedure was repeated. The experimenter judged the accuracy and precision of the calibration from the calibration plot, and chose to either proceed with the experimental tasks, or to re-run the five-point calibration.

Accuracy and precision were calculated during the gaze-contingent fixation stimulus that preceded each trial. Because the fixation stimulus was always at a known location, and because the trial would not begin until that location was fixated, we can use it to calculate the spatial error between the true gaze location and the gaze location reported by the eye tracker. Accuracy was calculated as the root-mean-square (RMS) of the euclidean distance between the location of each gaze sample and the location of the fixation stimulus. Precision was calculated as the RMS of the euclidean distance between each gaze sample and the centroid of all gaze samples.

### 1.3.1. Gap-overlap task

#### 1.3.1.1. Stimulus presentation

The gap-overlap task [(Elsabbagh et al., 2009, 2013)](https://www.zotero.org/google-docs/?XYZsEm) measures the efficiency of shifts in attention from a central to a peripheral stimulus under competition and non-competition conditions. Trials were presented in blocks of 12. All stimuli were presented at a size of 3cm x 3cm (2.86° x 2.86° at 60cm viewing distance). Reward stimuli were either a star, a sun, a dog, cat, pig, tiger or tortoise which were animated and accompanied by a sound. Each trial started with the onset of a central stimulus (CS), a cartoon image of an analogue clock accompanied by an alerting sound. After a 200ms period had elapsed, the peripheral stimulus (PS) was presented. In the baseline condition the CS was removed from the screen when the PS was presented. In the overlap condition the CS continued to be presented for the duration of the rest of the trial. In the gap condition the CS was removed from the screen and the PS was presented after a short gap. The PS was a cartoon cloud that appeared on either the left or the right side of the screen and was accompanied by a sound, 3cm (2.86°) from the edge, rotating at 500° per second until fixated by the participant. A reward stimulus was then presented at the location of the PS for 1000ms.

Gap/overlap trials were presented in blocks of 12. Within these, four baseline, gap and overlap trials were presented, two of each on the left of the screen, and two on the right. The order of trials within a block was randomised, but with the constraints that no more than three trials in a row could be to the same side, or of the same condition. This same presentation order and logic was applied equally across all blocks and for all participants. Four blocks of twelve trials were initially presented, and in cases where online validation reported fewer than 12 valid trials per condition, an additional fifth block was presented.

#### 1.3.1.1 Data extraction:

Mean saccadic reaction times (SRTs) were initially calculated for the Gap, Overlap and Baseline conditions. These were computed separately, using only valid trials. Participants with fewer than six valid trials-per-condition were removed entirely from the analysis. We then computed Gap Disengagement (Overlap-SRT minus Baseline-SRT) which, in addition to the Baseline condition, were the variables of interest in our analyses.

### 1.3.2 Popout task

#### 1.3.2.1 Stimulus presentation:

The face pop-out task [(Gliga et al., 2009)](https://www.zotero.org/google-docs/?Kk9a9C) involves the infant viewing a series of slides containing a face, scrambled face, car, bird and phone while their gaze direction and duration is measured. Infants were presented with a series of six annular visual arrays each composed of five objects in different locations on the screen (Gliga et al., 2009; Hendry, Jones, Bedford, Gliga, Charman, Johnson, et al., 2018). Each array contained: 1) a face with direct gaze; 2) a visual ‘noise’ image generated from the same face presented within the array by randomising the phase spectra of the face whilst keeping the amplitude and colour spectra constant to act as a control for the low-level visual properties of the face stimuli (Halit et al., 2004); 3) a bird; 4) a car; and, 5) a mobile phone. Each array was presented for 10 seconds and counter-balanced for the location of the face in the array. The stimulus array was presented full-screen with adjustments for a proper aspect ratio, at 43.8cm x 28.6cm (39.0° x 26.8° @ 60cm). The convex hull of the popout array had a diameter of 26.8° at 60cm viewing distance. The individual elements of the array were not all of the same width and height due to differences in the underlying shape of the object they depicted (for example, the phones were taller and narrow than the cars). The longest dimension of each array was maintained between 7.8° and 9.6° (at 60cm) for all stimuli. The shortest dimension was calculated against the longest dimension to maintain a correct aspect ratio. For each element of the array, the AOI mask was formed by colouring each non-background pixel, then dilating the mask by 2° of visual angle.

#### 1.3.2.2 Data extraction:

Areas-of-interest (AOIs) masks were placed around each stimulus. Each sample of gaze was converted into a logical vector of “AOI scores”, marked as 1 when gaze fell inside the AOI for a particular sample, and 0 where it did not. We then “interpolated” (or, more accurately, filled in) samples in this vector where the gaze data was missing. To ensure that we do not erroneously assign missing data to one AOI when in fact gaze during the missing period moved to another AOI, we only do this for a) runs of missing data where the valid data on either side of the missing run was to the same AOI, and b) runs with a duration <200ms. Wherever we report Proportion Valid Samples we use the value produced after interpolation. For each AOI, the proportion of samples within it was calculated by *number of samples in AOI / number of valid (non-missing) samples* (after interpolation)*.* Contiguous runs of samples within an AOI were identified and minimum/peak/mean look durations calculated. The mean proportion looking time to each AOI and minimum/peak/mean look values were calculated across valid trials only. Each AOI was scored by counting the number of samples of gaze data that fell on each AOI. Trials were marked as invalid if either a) the proportion of valid (non-missing) samples was less than 25%, or b) the duration of data was less than 5s. Participants with fewer than four valid trials were excluded from analysis.

Figure S1: Consort diagram of number of participants for each measure at each time point. Please note that whilst participants were seen at 2 years, this data is not reported in the current paper. Percentages indicate the retention rate; total number of possible visits differs between visits as participants could enter the study at different time points (between 5 and 14 months). As such, the total possible visits are as follows: 5 months = 110, 10 months = 160, 14 months to 3 years = 164.

#

# 2. Results

We ran a number of control analyses covarying variables that may impact our results.

## 2.1 Analysis 1: Controlling for eye tracking accuracy

Sleep deprivation can impact pupil reactivity (Wilhelm et al., 1998), which could then theoretically impact our eyetracking findings. Whilst we found no concurrent relationships between Sleep at any age point and the corresponding visual attention tasks, we thought it prudent to include metrics of eyetracking accuracy in our cross-lagged path models. We controlled for both the Accuracy and Precision (at each time point) in our Sleep and Visual Attention SEM models for the Popout, with model fit statistics indicating a ‘good’ fit [*χ^2^* (32) = 22.95, p = .74; CFI = 1, RMSEA = 0, SRMR = .06].

*Social attention in static stimuli (Popout task)*

Mean proportion of Face Looking was stable between 10- and 14-months (*β*=.42, p< .001), but not 5- to 10-months (*β*=.1, p=.37). As Night Sleep scores at 5-months decreased, mean proportion of Looking at the Face increased at 10-months (*β*=-.28, p=.01). In comparison, as Night Sleep scores at 10-months increased, so did the mean proportion of Face Looking at 14-months (*β*=.21, p=.06), though this was at a marginally significant level.

As the pattern of results do not change, we believe the findings in the main analyses are not due to effects of sleep-related pupil reactivity on data quality.

## 2.2 Analysis 2: Individual variables

When examining sleep metrics separately, we found a comparable pattern of results to that presented in the main text (though typically weaker). Specifically, Night Awakenings reduced with Age [F(2, 205) = 6.88, p = .001, η_p_^2^ = .06] and ASD-L infants had an increased number of awakenings [F(1, 139) = 4.79, p = .03, η_p_^2^ = .03]. To examine the ASD*ADHD-L*Age interaction [F(2, 205) = 5.4, p = .005, η_p_^2^ = .05], we conducted univariate ANOVAs for each age group and found that participants with an elevated likelihood for ASD woke more frequently during the night at 14 months [F(1, 118) = 11.61, p = .001, η_p_^2^ = .09], but not at 5 [F(1, 98) = .003, p = .95, η_p_^2^ = .0] or 10 months [F(1, 121) = 2.04, p = .16, η_p_^2^ = .02].

We found that ISOP scores increased with Age [F(2, 301) = 14.3, p < .001, η_p_^2^ = .09]. To examine the significant interaction of ASD-L*Age [F(2, 301) = 4.25, p = .02, η_p_^2^ = .09], we conducted univariate ANOVAs for each age group and found that participants with an elevated likelihood for ASD had more problems settling to sleep at 10 [F(1, 127) = 7.05, p = .009, η_p_^2^ = .05] and 14 months [F(1, 128) = 4.82, p = .03, η_p_^2^ = .04], but not at 5 months [F(1, 103) = 1.79, p = .18, η_p_^2^ = .02].

With respect to ASD outcome (ASD+/ASD-), we found increased sleep onset problems in the ASD- group [F(1, 91) = 7.27, p = .008, η_p_^2^ = .07]. To investigate the significant interaction of Outcome and Age [F(2, 205) = 5.67, p = .004, η_p_^2^ = .05], we conducted pairwise comparisons. Here we found that the ASD- group demonstrated increased ISOP scores from 5 to 10 months (mean diff = 3.29, df = 224, p < .001, CI = -4.51 to -2.1), with increases between 5 and 14 months (mean diff = 3.86, df = 228, p < .001, CI = -5.07 to -2.64) but no difference between 10 and 14 months (mean dif = .57, df = 197, p = .33, CI = -1.7 to .57). In comparison, the ASD+ showed no change in ISOP scores between any age point (5 vs 10 months: mean dif = 2.09, df = 215, p = .24, CI = -1.4 to 5.6; 5 vs 14 months: mean dif = 1.64, df = 205, p = .35, CI = -1.81 to 5.08; 10 vs 14 months: mean dif = .46, df = 192, p = .8, CI = -3.93 to 3.02) (Bonferroni corrected to p < .008).

The ASD+ group woke more frequently during the night than the ASD- group [F(1, 109) = 3.13, p = .08, η_p_^2^ = .03] at a trend level. We found no group differences in Night Sleep Duration [F(1, 109) = 1, p = .31, η_p_^2^ = .001] .

When examining the longitudinal associations between our individual Night Sleep variables and visual attention and ASD traits, we found comparable results to that in the main analyses with our Night Sleep Factor. Overall, the pattern of beta coefficients for the association between individual sleep variables at 5 months and visual attention at 10 months/ SRS scores at 3 years were consistent with the associations seen with the Night Factor scores, though were generally weaker (Night sleep factor and visual attention at 10 months β =-.3 p = .005; Night Sleep duration and visual attention at 10 months; β = .02, p = .85; Night awakenings and visual attention at 10 months: β = .14, p = .25; ISOP score and visual attention at 10 months: β = -.18, p = .18; Night sleep factor and SRS at 3 years: β = .44, p = <.001; Night Sleep duration and SRS at 3 years; β = .2, p = .13; Night awakenings and SRS at 36 months: β = -.15, p = .22; ISOP score and SRS at 36 months: β = -.01 . p = .93).

## 2.3 Analysis 3: Confirmatory Factor Analysis

Due to our findings regarding age related changes in the relation between our Night Sleep factor and other phenotypes, we investigated this factor over time. We conducted a confirmatory factor analysis (lavaan) using variables that loaded >.3 on the Night Sleep Factor (Night Sleep, Night Awakenings, ISOP and Day Naps) and Day Sleep Factor (Day Naps, Day Sleep, ISOP). This provided a good fit to the data (X2(2)=5.7, p=0.058; CFI=.969, RMSEA=0.075). Within a multigroup CFA we tested the change in fit if paths were unconstrained across timepoints. This resulted in no convergence for a model varying the loading of Night Sleep on the night factor; worsening fit for varying the loading of Night Awakenings (χ^2^ = 44.53 vs 40.03); and no significant change in fit for Day Naps (χ^2^(2)=1.62, p = 0.045). For the Day Factor, fit was worse with unconstrained loadings for Day Sleep (χ^2^ = 40.69 vs 40.03); and would not converge with unconstrained loadings for Day Naps. Varying the loading of ISOP on the Night Sleep Factor resulted in a significant improvement in the model (χ^2^(2) = 10.96, p = 0.005), with a positive loading at 5 months (z=2.8, p = 0.005), no significant loading at 10 months (z=1.6, p = 0.1) and a negative loading at 14 months (z=-3.1, p = 0.002). Varying the loading of ISOP on the Day Sleep Factor resulted in a significant improvement in the model (χ^2^(2) = 10.7, p = 0.005), with no significant loading at 5 months (z=.397, p = 0.69) or 10 months (z=1.38, p = 0.20) and a negative loading at 14 months (z=-4.3, p <0.001).

Since the ISOP appeared to change its loading on both Night and Day Sleep Factors with time, we repeated the analyses that showed change in relations over time (particularly between sleep and SRS scores, and sleep and visual attention) with Factor Scores computed omitting the ISOP. This showed the comparable result that increased Night Sleep scores at 10 months associated with increased Face Looking at 14 months (β =.3, p = .06), though at a marginally significant level. However, there was no longer an association between Night Sleep at 5 months and Face looking at 10 months (β =.13, p = .33). In terms of the associations between Night Sleep and later SRS traits, we find the same pattern of results as with the original factor scores; increased Night Sleep scores at 5 months associated with more ASD traits at 3 years (β =.26, p = .05), whilst decreased Night Sleep at 14 months was associated with increased ASD traits at 3 years (β =.39, p = .004). Thus, this developmental reversal is unlikely to be related to changes in the meaning of factor scores over time.

In addition to this, we reconstructed our sleep factor scores allowing for the ISOP loadings to vary by age. Here we found that the model fit for the SEM approach declined when using this approach [χ^2^ (4) = 9.74, p = .04, CFI = .98, RMSEA = .09, SRMR = .04] versus [χ^2^ (4) = 2.06, p = .73, CFI = 1, RMSEA = .0, SRMR = .03] for our original factors. This indicates that using age-adjusted factors provides a less good capture of variance in the dataset.

## 2.4 Analysis 4: Total Sleep Duration

To determine whether total sleep duration or sleep efficiency would provide additional information we conducted additional analyses with Total Sleep Duration (Day Sleep Duration + Night Sleep Duration) and Percentage of Night Sleep (Night Sleep Duration/Total Sleep Duration). Of note, these variables were significantly correlated with the original Night Sleep Factor [r(332) = .65, p < .001 and r(332) = .26, p < .001 respectively].

We found that Total Sleep Duration increased with Age [F(2, 217) = 3.2, p = .04, η_p_^2^ = .03], with the ASD-L group sleeping less than those without a family history of ASD [F(1, 144) = 7.14, p = .008, η_p_^2^ = .05]. We found that the Percentage of Night Sleep increased with Age [F(2, 229) = 3.26, p = .04, η_p_^2^ = .03], but did not vary by ASD likelihood [F(1, 144) = .23, p = .64, η_p_^2^ = .002]. Neither Total Sleep Duration [F(1, 109) = .31, p = .58, η_p_^2^ = .003] or Percentage of Night Sleep [F(1, 116) = .09, p = .76, η_p_^2^ = .001] were related to ASD Outcome. We found no significant associations between Total Sleep Duration/Efficiency and visual attention (all ps > .15).

Thus, it does not appear that metrics of total sleep duration or sleep efficiency provide clearer associations with phenotype in this sample

## 2.5 Analysis 5: Concurrent relationships between Night Sleep and ASD symptoms

Given the association between Night Sleep scores at 14 months and later ASD symptoms at 3 years, we also examined the relationship between Sleep and concurrent ASD symptoms at 14 months of age. We used the Autism Observation Score for Infants (Bryson et al., 2008) which is a standardised research assessment that examines ASD traits in infants in the first two years of life. The measure involves a semi-structured play session between a researcher and the infant, examining factors such as social-communicative development, atypical sensorimotor behaviours and repetitive behaviours). Higher scores on this measure indicate greater ASD traits.

Bivariate correlations showed no association between Night Sleep Scores at 14 months and AOSI total scores at 14 months [r(97) = -.04, p = .7].

## 2.6 Analysis 6: ISOP score control analyses

We further examined the ISOP score, with follow-up analyses indicating that the ISOP score was only differentially related to other aspects of Night Sleep at 5 months, and thus any difficulty in interpretation is restricted to that timepoint and that measure. For example, raw correlations indicate that the ISOP did not associate as strongly with overall sleep factor scores at 5 months but did at older ages (correlations of r=.15, p=.15 at 5m, r=.65, p<0.001 at 10m, and at 14m r=.74, p<0.001). Consistent with these observations, there was a negative correlation between ISOP scores at 5 and 10 months such that higher scores (more problems settling) at 5 months were associated with lower scores (fewer problems settling) at 10 months (r = -.58, p < .001). Further, at the level of raw measures at 10 and 14 months higher ISOP scores (more problems settling) associated with fewer Night Awakenings (10 months: r = -.46, p < .001; 14 months: r = -.53, p < .001) and lower Night Sleep (10 months: r = .28, p = .002; 14 months: r = .36, p < .001). However, the reverse was true at 5 months such that more problems settling associated with increased Night Awakenings (r = .37, p < .001) and shorter Night Sleep at a trend level (r = -.17, p = .1). Thus, the overall pattern indicates that the ISOP score associates differently with other elements of sleep at 5 months than at 10 and 14 months. This issue does not affect the majority of our results, which pertain to the 10 and 14-month timepoints. Further, the issue only affects the 5-month timepoint to the extent that the contribution of the ISOP to factor scores at that age (with the lowest loading) imperfectly reflects its underlying relationship to the three other sleep measures.

Due to increased measurement variance in the ISOP score at the 5 month timepoint (see SM 2.3), we conducted a number of control analyses. Specifically, we first conducted SEM models where we removed data from the 5-month time point. Here, we found comparable results to those presented in the original models; Night Sleep factor scores are stable between 10 and 14 months of age (β = .72, p < .001), with increased Night Sleep scores at 10 months associating with increased looking at Static Social stimuli at 14 months (β = .33, p = .04). Additionally, we found that decreased Night Sleep scores at 14 months associated with increased ASD traits at 3 years (SRS: β = -.51, p <.001). Increased Night Sleep scores at 14 months was also associated with increased cognitive (MSEL: β = .34, p = .02) and socialisation skills (VABS: β = .34, p = .03).

Additionally, our cross lagged model shows a significant association between our Night Sleep Factor at 5 and 10 and 10 and 14 months of age (β = .33, p =.002, β = .72, p <.001). The magnitude of associations between the individual sleep measures with age are similar (5 to 10 months: Night sleep duration: β = .44, p < .001; Night awakenings: β = .4, p <.001; ISOP: β = -.56, p <.001; 10 to 14 months Night sleep duration: β = .46, p < .001; Night awakenings: β = .74, p <.001; ISOP: β = .58, p <.001).

Finally, the key autism-related results are also replicated if we construct factor scores without the ISOP (increased Night Sleep scores at 5 months associated with more ASD traits at 3 years (β =.26, p = .05, whilst decreased Night Sleep at 14 months was associated with increased ASD traits at 3 years; β =.39, p = .004).

Taken together, though the ISOP score was only differentially related to other aspects of Night Sleep at 5 months, the inclusion/exclusion of this variable in our Night Sleep Factor does not change the pattern of results that are observed in the main text.

# 3. Tables and figures referred to in the main text

## 3.1 PCA factor loadings for Day and Night Sleep

| Table S3: Table showing factor loadings for each sleep variable from the SSQ and IBQ-R | | |
| --- | --- | --- |
|  | Night sleep factor | Day sleep factor |
| Frequency of night awakenings | -.78 | -.29 |
| Night sleep duration | .63 | .16 |
| Frequency of day naps | -.53 | .44 |
| Day sleep duration | .17 | .84 |
| Sleep onset problems score | .6 | -.4 |

## 3.2 Bivariate correlations between individual sleep variables

| Table S4: Table showing correlations between individual variables | | | | |
| --- | --- | --- | --- | --- |
|  | Naps | Night Sleep duration | Awakenings | ISOP |
| Day Sleep duration | .07 | .06 | -.23 | -.07 |
|  | .22 | .24 | <.001 | .18 |
| Naps |  | -.09 | .22 | -.23 |
|  |  | .11 | <.001 | <.001 |
| Night Sleep duration |  |  | -.37 | .19 |
|  |  |  | <.001 | <.001 |
| Awakenings |  |  |  | -.24 |
|  |  |  |  | <.001 |

## 3.3. Cross lagged models between Sleep, Visual Attention and later traits

| **Table S5: Table showing beta coefficient, p values and fit statistics for crossed lagged models** | | |
| --- | --- | --- |
| **Night Sleep + Visual attention** | | |
| **Model 1: Sleep and Attention Shifting (Gap disengagement)**  **Fit statistics:** *χ^2^* (4) = 2.06, p = .73, CFI = 1, RMSEA = .0, SRMR = .03 | | |
| **Path** | **beta** | **P value** |
| Night sleep 5 months 🡪 Gap disengagement 5 months | β = -.13 | .26 |
| Night sleep 5 months 🡪 Gap disengagement 10 months | β = .07 | .55 |
| Gap disengagement 5 months 🡪 Night sleep 10 months | β = -.001 | .99 |
| Night sleep 10 months 🡪 Gap disengagement 10 months | β = -.13 | .2 |
| Night sleep 10 months 🡪 Gap disengagement 14 months | β = .08 | .63 |
| Gap disengagement 10 months 🡪 Night sleep 14 months | β = -.04 | .58 |
| Night sleep 14 months 🡪 Gap disengagement 14 months | β = -.04 | .77 |
| Gap disengagement 5 months 🡪 Gap disengagement 10 months | β = .25 | .03 |
| Gap disengagement 10 months 🡪 Gap disengagement 14 months | β = .43 | <.001 |
| **Model 2: Sleep and Visual Attention**  **Fit statistics:** *χ^2^* (4) = 1.82, p = .77; CFI = 1; RMSEA = .0; SRMR = .03 | | |
| **Path** | **beta** | **P value** |
| Night sleep 5 months 🡪 Popout (Face) 5 months | β = -.09 | .38 |
| Night sleep 5 months 🡪 Popout (Face) 10 months | β = -.3 | .005 |
| Popout (Face) 5 months 🡪 Night sleep 10 months | β = -15 | .2 |
| Night sleep 10 months 🡪 Popout (Face) 10 months | β =.08 | .5 |
| Night sleep 10 months 🡪 Popout (Face) 14 months | β = -.34 | <.001 |
| Popout (Face) 10 months 🡪 Night sleep 14 months | β = -.07 | .45 |
| Night sleep 14 months 🡪 Popout (Face) 14 months | β = -.18 | .24 |
| Popout (Face) 5 months 🡪 Popout (Face) 10 months | β = .14 | .22 |
| Popout (Face) 10 months 🡪 Popout (Face) 14 months | β = .4 | <.001 |
| **Model 3: Sleep, Visual Attention and ASD traits**  **Fit statistics:** *χ^2^* (4) = 2.15, p = .71; CFI = 1; RMSEA = .0; SRMR = .03 | | |
| **Path** | **beta** | **P value** |
| Night sleep 5 months 🡪 SRS Total 3 years | β = .44 | <.001 |
| Night sleep 10 months 🡪 SRS Total 3 years | β = -.01 | .8 |
| Night sleep 14 months 🡪 SRS Total 3 years | β = -.48 | .001 |
| Popout (Face) 5 months 🡪 SRS Total 3 years | β = -.16 | .12 |
| Popout (Face) 10 months 🡪 SRS Total 3 years | β = .26 | .02 |
| Popout (Face) 14 months 🡪 SRS Total 3 years | β = .05 | .61 |
| **Model 4: Sleep, Visual attention and Socialisation abilities**  **Fit statistics:** *χ^2^* (4) = 1.58, p = .81; CFI = 1; RMSEA = .0; SRMR = .02 | | |
| **Path** | **beta** | **P value** |
| Night sleep 5 months 🡪 VABS socialisation 3 years | β = -.2 | .1 |
| Night sleep 10 months 🡪 VABS socialisation 3 years | β = .12 | .48 |
| Night sleep 14 months 🡪 VABS socialisation 3 years | β = .34 | .03 |
| Popout (Face) 5 months 🡪 VABS socialisation 3 years | β = .1 | .37 |
| Popout (Face) 10 months 🡪 VABS socialisation 3 years | β = -.05 | .67 |
| Popout (Face) 14 months 🡪 VABS socialisation 3 years | β = -.28 | .01 |
| **Model 5: Sleep, Visual attention and cognitive abilities**  **Fit statistics:** χ^2^ (4) = 1.86, p = .76; CFI = 1; RMSEA = .0; SRMR = .02 | | |
| **Path** | **beta** | **P value** |
| Night sleep 5 months 🡪 MSEL ELC 3 years | β = -.1 | .45 |
| Night sleep 10 months 🡪 MSEL ELC 3 years | β = -.14 | .34 |
| Night sleep 14 months 🡪 MSEL ELC 3 years | β = .3 | .03 |
| Popout (Face) 5 months 🡪 MSEL ELC 3 years | β = .17 | .12 |
| Popout (Face) 10 months 🡪 MSEL ELC 3 years | β = -.3 | .01 |
| Popout (Face) 14 months 🡪 MSEL ELC 3 years | β = -.02 | .82 |

## 3.4 Scatter plots of Night Sleep and later traits


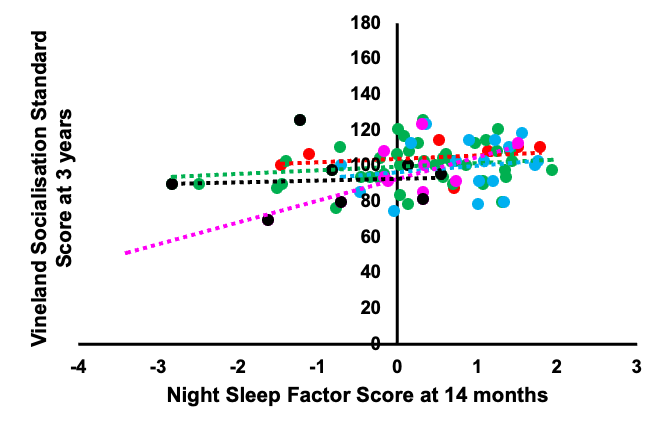

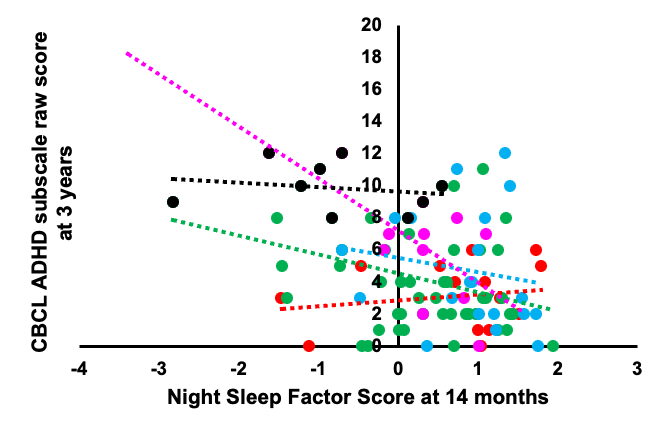

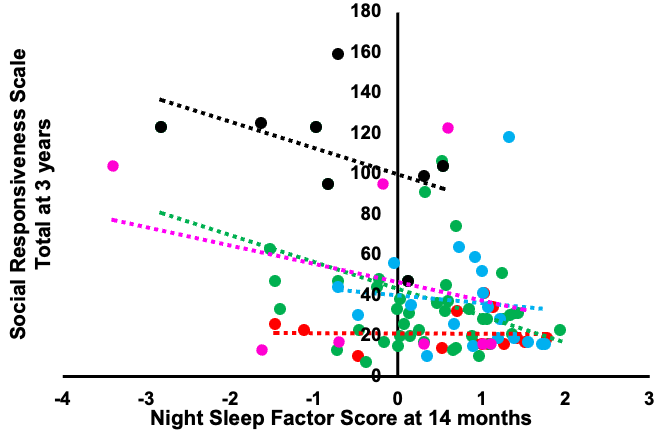


(A)

(B)


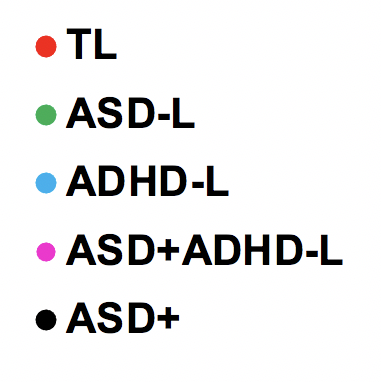


(C)

Figure S2: Scatter graphs showing Night Sleep Factor at 14 months against SRS Total (Panel A), VABS Socialisation (Panel B) and CBCL ADHD (Panel C) across elevated likelihood and ASD+ groups

# 4. References

|  |
| --- |

Bryson, S. E., Zwaigenbaum, L., McDermott, C., Rombough, V., & Brian, J. (2008). The Autism Observation Scale for Infants: scale development and reliability data. *Journal of autism and developmental disorders*, 38(4), 731-738.

Conners, C. K. (2008). Conners third edition (Conners 3). *Los Angeles, CA: Western Psychological Services*

Conners, C. K., & Goldstein, S. (2009). *Conners Early childhood: Manual*. Toronto, ON: Multi-Health Systems Incorporated.

Constantino, J. N., & Gruber, C. P. (2012). *Social responsiveness scale: SRS-2*. Torrance, CA: Western Psychological Services.

Elsabbagh, M., Volein, A., Holmboe, K., Tucker, L., Csibra, G., Baron‐Cohen, S., ... & Johnson, M. H. (2009). Visual orienting in the early broader autism phenotype: disengagement and facilitation. *Journal of Child Psychology and Psychiatry*, *50*(5), 637-642.

Elsabbagh, M., Fernandes, J., Webb, S. J., Dawson, G., Charman, T., Johnson, M. H., & British Autism Study of Infant Siblings Team. (2013). Disengagement of visual attention in infancy is associated with emerging autism in toddlerhood. *Biological psychiatry*, *74*(3), 189-194.

Gliga, T., Elsabbagh, M., Andravizou, A., & Johnson, M. (2009). Faces attract infants' attention in complex displays. *Infancy*, *14*(5), 550-562.

Goodman, R., Ford, T., Richards, H., Gatward, R., & Meltzer, H. (2000). The Development and Well-Being Assessment: description and initial validation of an integrated assessment of child and adolescent psychopathology. *The Journal of Child Psychology and Psychiatry and Allied Disciplines*, *41*(5), 645-655.

Halit, H., Csibra, G., Volein, A., & Johnson, M. H. (2004). Face‐sensitive cortical processing in early infancy. *Journal of Child Psychology and Psychiatry*, *45*(7), 1228-1234.

Hendry, A., Jones, E. J., Bedford, R., Gliga, T., Charman, T., & Johnson, M. H. (2018). Developmental change in look durations predicts later effortful control in toddlers at familial risk for ASD. *Journal of Neurodevelopmental Disorders*, *10*(1), 1-14.

Jones, E. J. H, Mason, L., Begum Ali, J., Van Den Boomen, C., Braukmann, R., Cauvet, E., ... & Johnson, M. H. (2019). Eurosibs: Towards robust measurement of infant neurocognitive predictors of autism across Europe. *Infant Behavior and Development*, *57*, 101316.

Lord, C., Rutter, M., & Le Couteur, A. (1994). Autism Diagnostic Interview-Revised: a revised version of a diagnostic interview for caregivers of individuals with possible pervasive developmental disorders. *Journal of autism and developmental disorders*, *24*(5), 659-685.

Lord, C., Rutter, M., DiLavore, P., Risi, S., Gotham, K., & Bishop, S. (2012). Autism diagnostic observation schedule–2nd edition (ADOS-2). *Los Angeles, CA: Western Psychological Corporation*, *284*

MacDuffie, K. E., Shen, M. D., Dager, S. R., Styner, M. A., Kim, S. H., Paterson, S., ... & Estes, A. M. (2020). Sleep onset problems and subcortical development in infants later diagnosed with autism spectrum disorder. *American Journal of Psychiatry, 177*(6), 518-525.

Miller, M., Iosif, A. M., Bell, L. J., Farquhar-Leicester, A., Hatch, B., Hill, A., ... & Ozonoff, S. (2020). Can familial risk for ADHD be detected in the first two years of life?. *Journal of Clinical Child & Adolescent Psychology*, 1-13.

|  |
| --- |

Mullen, E. M. (1995). *Mullen scales of early learning* (pp. 58-64). Circle Pines, MN: AGS.

Russell, G., Rodgers, L. R., Ukoumunne, O. C., & Ford, T. (2014). Prevalence of parent-reported ASD and ADHD in the UK: findings from the Millennium Cohort Study. *Journal of autism and developmental disorders*, *44*(1), 31-40.

Rutter, M., Bailey, A., & Lord, C. (2003). *The social communication questionnaire: Manual*. Western Psychological Services.

Simonoff, E., Pickles, A., Charman, T., Chandler, S., Loucas, T., & Baird, G. (2008). Psychiatric disorders in children with autism spectrum disorders: prevalence, comorbidity, and associated factors in a population-derived sample. *Journal of the American Academy of Child & Adolescent Psychiatry*, *47*(8), 921-929.

Sparrow, S.S., Cicchetti, D.V., & Balla, D.A. (2005). Vineland-II adaptive behavior scales, second edition, survey forms manual. Bloomington, MN: PsychCorp.

Võ, M. L. H., Smith, T. J., Mital, P. K., & Henderson, J. M. (2012). Do the eyes really have it? Dynamic allocation of attention when viewing moving faces. *Journal of vision*, *12*(13), 3-3.

Wilhelm, B., Wilhelm, H., Lüdtke, H., Streicher, P., & Adler, M. (1998). Pupillographic assessment of sleepiness in sleep-deprived healthy subjects. *Sleep, 21*(3), 258-265.

# 5. Appendix

Appendix A: Table of missing data across tasks

| **Task** | **Reason** | **TL** | **ASD-L** | **ADHD-L** | **ASD+ADHD-L** |
| --- | --- | --- | --- | --- | --- |
| 5mo Gap Baseline | Trials | 1 | 7 | 2 |  |
|  | Tech | 1 | 3 | 1 |  |
| 5mo Gap Disengagement | Trials | 2 | 8 | 2 | 1 |
|  | Tech | 1 | 3 | 1 |  |
| 10mo Gap Baseline | Trials | 1 | 7 |  |  |
|  | Tech |  | 1 |  | 1 |
|  | Visit | 2 | 3 | 1 |  |
| 10mo Gap Disengagement | Trials | 1 | 8 | 1 |  |
|  | Tech |  | 1 |  | 1 |
|  | Visit | 2 | 3 | 1 |  |
| 14mo Gap Baseline | Trials |  | 4 | 1 | 2 |
|  | Tech |  |  |  |  |
|  | Visit | 3 | 7 | 7 | 1 |
| 14mo Gap Disengagement | Trials |  | 4 | 1 | 2 |
|  | Tech |  |  |  |  |
|  | Visit | 3 | 7 | 7 | 1 |
| 5mo Popout Face/Overall looking | Trials |  | 1 |  |  |
|  | Tech | 1 | 2 | 1 |  |
|  | Visit |  | 1 |  |  |
| 10mo Popout Face/Overall looking | Trials |  | 1 | 2 |  |
|  | Tech | 1 | 2 | 1 | 2 |
|  | Visit | 2 | 2 | 1 |  |
|  | Task | 2 | 3 | 1 |  |
|  | Infant |  | 4 | 1 | 1 |
| 14mo Popout Face/Overall looking | Trials |  | 1 | 1 |  |
|  | Tech |  | 1 |  |  |
|  | Visit | 6 | 7 | 7 | 1 |
|  | Task |  | 2 |  |  |
|  | Infant | 1 | 4 | 1 | 3 |
| 10mo 50 Faces | Trials | 1 | 6 | 3 |  |
|  | Tech |  |  |  | 2 |
|  | Visit | 2 | 2 | 1 |  |
|  | Task | 2 | 3 | 1 |  |
|  | Infant |  | 13 | 3 | 5 |
| 14mo 50 Faces | Trials |  | 2 | 1 |  |
|  | Tech |  | 1 |  |  |
|  | Visit | 6 | 7 | 8 | 1 |
|  | Task |  | 2 |  |  |
|  | Infant | 1 | 8 |  | 4 |
| 5mo SSQ | No return | 2 |  | 2 |  |
|  | Partial | 1 | 4 | 2 |  |
| 10mo SSQ | No return | 5 | 11 | 4 | 2 |
|  | Partial | 1 | 4 | 2 | 1 |
|  | Visit | 2 | 2 | 1 | 1 |
| 14mo SSQ | No return | 4 | 7 | 4 | 2 |
|  | Partial |  | 8 | 3 | 1 |
|  | Visit | 6 | 5 | 5 | 1 |
| 5mo IBQ | No return | 3 |  | 3 |  |
|  | Partial |  | 3 |  |  |
| 10mo IBQ | No return | 5 | 14 | 3 | 2 |
|  | Partial |  | 2 |  |  |
|  | Visit | 2 | 2 |  |  |
| 14mo IBQ | No return | 2 | 7 | 4 | 1 |
|  | Partial | 1 | 1 |  |  |
|  | Visit | 6 | 5 | 5 | 1 |

Trials = Not enough valid trials/data; Tech = tech issues; Visit = did not attend session/dropped out; Task = task not administered due to time constraints/parental wishes; Infant = fussed out; No return = questionnaires not returned; Partial = only partial completion so could not be used in analyses
